# Gene expression profiles in testis of pigs with extreme high and low levels of androstenone

**DOI:** 10.1186/1471-2164-8-405

**Published:** 2007-11-07

**Authors:** Maren Moe, Theo Meuwissen, Sigbjørn Lien, Christian Bendixen, Xuefei Wang, Lene Nagstrup Conley, Ingunn Berget, Håvard Tajet, Eli Grindflek

**Affiliations:** 1 The Norwegian Pig Breeders Association (NORSVIN), Hamar, Norway.; 2 Department of Animal and Aquacultural Sciences, Norwegian University of Life Sciences, Ås, Norway.; 3 Centre for Integrative Genetics (CIGENE), Norwegian University of Life Sciences, Ås, Norway.; 4 Faculty of Agricultural Sciences, University of Aarhus, Tjele, Denmark.; 5 MATFORSK, Osloveien 1, Ås, Norway.

## Abstract

**Background:**

Boar taint is a major obstacle when using uncastrated male pigs for swine production. One of the main compounds causing this taint is androstenone, a pheromone produced in porcine testis. Here we use microarrays to study the expression of thousands of genes simultaneously in testis of high and low androstenone boars. The study allows identification of genes and pathways associated with elevated androstenone levels, which is essential for recognising potential molecular markers for breeding purposes.

**Results:**

Testicular tissue was collected from 60 boars, 30 with extreme high and 30 with extreme low levels of androstenone, from each of the two breeds Duroc and Norwegian Landrace. The samples were hybridised to porcine arrays containing 26,877 cDNA clones, detecting 563 and 160 genes that were differentially expressed (p < 0.01) in Duroc and Norwegian Landrace, respectively. Of these significantly up- and down-regulated clones, 72 were found to be common for the two breeds, suggesting the possibility of both general and breed specific mechanisms in regulation of, or response to androstenone levels in boars. Ten genes were chosen for verification of expression patterns by quantitative real competitive PCR and real-time PCR. As expected, our results point towards steroid hormone metabolism and biosynthesis as important biological processes for the androstenone levels, but other potential pathways were identified as well. Among these were oxidoreductase activity, ferric iron binding, iron ion binding and electron transport activities. Genes belonging to the cytochrome P450 and hydroxysteroid dehydrogenase families were highly up-regulated, in addition to several genes encoding different families of conjugation enzymes. Furthermore, a number of genes encoding transcription factors were found both up- and down-regulated. The high number of clones belonging to ferric iron and iron ion binding suggests an importance of these genes, and the association between these pathways and androstenone levels is not previously described.

**Conclusion:**

This study contributes to the understanding of the complex genetic system controlling and responding to androstenone levels in pig testis. The identification of new pathways and genes involved in the biosynthesis and metabolism of androstenone is an important first step towards finding molecular markers to reduce boar taint.

## Background

The production of uncastrated male pigs is profitable to the swine production, because it leads to improved feed efficiency and greater lean yield of the carcass [1]. However, production of entire males is a challenge due to boar taint, an unpleasant odour and flavour often present in the meat of un-castrated male pigs [2]. Castration is undesirable both for ethical as well as for economical reasons and thus alternative methods are needed to prevent tainted meat. Boar taint is primarily caused by high levels of the two components androstenone and/or skatole in the pig carcasses [3,4]. Moderate to high heritabilities have been shown for both androstenone and skatole levels in fat [5-7]. For example, a recent study of Duroc and Norwegian Landrace showed heritabilities ranging from 0.5–0.6 for androstenone and 0.23–0.56 for skatole [8].

Androstenone (5α-androst-16-en-3-one) is a 16-androstene steroid produced from pregnenolone in the steroid hormone pathway in boar testis near sexual maturity. It is released into the blood and transported to salivary glands [9], where it regulates reproductive functions in female pigs [10]. Due to its lipophilic properties, it is also easily stored in the adipose tissue [11], causing boar taint when the fat is heated. Another steroid produced from pregnenolone in the same pathway is testosterone. Testosterone stimulates growth and fertility and the challenge is to reduce the level of androstenone without affecting the level of testosterone. Genes specifically affecting the production of androstenone in the testis are therefore of interest, as well as genes involved in degradation and elimination of this steroid.

Several candidate genes have been suggested to affect levels of androstenone. The biosynthesis of 16-androstenes from pregnenolone is catalysed by the andien-β synthase enzyme system [12], which consists of cytochrome P450-C17 (CYP17) and cytochrome b5 (CYB5) [13]. Although *CYP17 *has been proposed as a very potent candidate gene affecting levels of androstenone, studies so far have not found significant effects of SNPs within the *CYP17 *gene and androstenone levels [14,15]. Similarly, it has not been possible to find a significant correlation between levels of CYP17 and levels of 16-androstene steroids in fat [14]. CYB5 has been found positively correlated to the production of androstenone [14]. In addition, a SNP in the 5' un-translated region of porcine *CYB5 *is found to be associated with decreased fat androstenone levels [16].

Another potent class of candidate genes is the sulphotransferase (SULT) enzymes whose main function is to transfer a sulfo group to a range of molecules including for example steroid hormones [17]. The SULTs have been proposed to regulate the amount of unconjugated 5α-androstenone available for accumulation in fat [18], and high proportions of sulphoconjugated to unconjugated 16-androstene steroids are observed in porcine plasma and Leydig cells [18,19].

The levels of androstenone vary between breeds, with e.g. higher androstenone levels in Duroc and Hampshire compared to Landrace and Yorkshire [20]. It is therefore of interest to investigate gene expression patterns in individuals with high or low levels of androstenone in different breeds. In this study boars with extreme levels of androstenone were selected from the two commercial breeding populations in Norway, Duroc and Norwegian Landrace, for gene expression analysis in testis using microarray technology. The objective of the study was to identify differentially expressed genes that could point towards pathways associated with extreme levels of androstenone in pigs. To our knowledge, this is the first microarray experiment performed in this context.

## Results

Porcine cDNA microarrays containing 26,877 clones were used to examine the transcript profile of Duroc (D) and Norwegian Landrace (NL) pigs with high (H) and low (L) levels of androstenone. A total of 1533 NL boars and 1027 D boars were analysed and average androstenone levels were 1.17 ± 1.15 μg/g and 3.22 ± 2.68 μg/g for NL and D, respectively. 30 high and 30 low androstenone boars were selected from each breed and average values were 5.91 ± 2.41 μg/g for NL high androstenone (NLH), 0.15 ± 0.04 μg/g for NL low androstenone (NLL), 10.27 ± 2.68 μg/g for D high androstenone (DH) and 0.42 ± 0.13 μg/g for D low androstenone (DL). Testicle derived samples were hybridised using a balanced block design and significantly affected genes were identified using linear models (Limma). Box plots (See Additional file 1: Boxplot of the arrays) show that the deviation of the raw log ratios from 0 was successfully normalised. The statistical test detected 563 and 160 clones as differentially expressed in D and NL, respectively (p < 0.01) (See Additional file 2: Microarray results analysed using Limma, Duroc and Additional file 3: Microarray results analysed using Limma, Norwegian Landrace). The top 100 genes are presented for D (Table 1) and NL (Table 2). Venn diagrams were created to explore any overlap between breeds and 72 genes were found in common for D and NL (See Additional file 4: Genes common for Duroc and Norwegian Landrace at p < 0.01). Another more robust, but less powerful non-parametric test for statistical analysis, namely Fisher's Sign Test (FST) was performed as well. (See Additional file 5: Microarray results analysed using Fisher's sign test, Duroc and additional file 6: Microarray results analysed using Fisher's sign test, Norwegian Landrace).

**Table 1:** Top 100 genes identified in Duroc

ID	Name	gene_id	M	adj.P.Val
211570	Similar to hypothetical protein (LOC475155)	XM_532385.1	0.746	4.28e-12
103463	Cytochrome P450 17A1 (CYP17A1)	NM_214428.1	1.227	6.05e-12
218005			1.100	8.28e-12
217877	Ferritin, light polypeptide (FTL)	NM_000146.3	1.033	1.27e-11
209883			0.550	1.29e-11
213434	Eukaryotic translation elongation factor 1 alpha 1 (EEF1A1)	NM_001402.4	1.084	1.29e-11
203529	Cystatin F (leukocystatin) (CST7)	NM_003650.2	0.726	1.57e-11
102025	Cytochrome b-5 (CYB5)	NM_001001770.1	0.529	1.57e-11
210231	Ferritin, light polypeptide (FTL)	NM_000146.3	1.032	1.57e-11
215500	Ferritin, light polypeptide (FTL)	NM_000146.3	0.986	5.24e-11
210851	Ferritin, light polypeptide (FTL)	NM_000146.3	1.006	6.23e-11
216813	Kallikrein 6 (neurosin, zyme) (KLK6), transcript variant C	NM_001012965.1	0.892	6.45e-11
211448	Golgi phosphoprotein 3-like (GOLPH3L)	NM_018178.3	0.927	1.55e-10
103177	Cytochrome b-5 (CYB5)	NM_001001770.1	0.768	9.43e-10
216525	Chromosome 22 open reading frame 16 (C22orf16)	NM_213720.1	0.639	1.52e-09
214052	Carbonyl reductase/NADP-retinol dehydrogenase (DHRS4)	NM_214019.1	0.889	1.64e-09
218092	Procollagen C-endopeptidase enhancer (PCOLCE)	NM_002593.2	0.606	1.69e-09
100650	Brain expressed, X-linked 1 (BEX1)	NM_018476.2	0.537	1.98e-09
216417	DNA helicase HEL308 (HEL308)	NM_133636.1	0.757	2.11e-09
217089	Chromosome 20 open reading frame 22 (C20orf22)	NM_015600.2	0.812	2.19e-09
211821	Phosphatidylserine receptor (PTDSR)	NM_015167.1	0.891	2.61e-09
213358	Microtubule-associated protein 1 light chain 3 alpha (MAP1LC3A)	NM_032514.2	0.811	3.78e-09
206784	Ferritin heavy-chain (FTH1)	NM_213975.1	0.478	4.72e-09
216513	Prostate androgen-regulated transcript 1 (PART1)	NM_016590.2	0.693	4.72e-09
101322	Ferredoxin (FDX1)	NM_214065.1	0.498	8.99e-09
215969	N-acetylglucosamine-1-phosphotransferase, gamma subunit (GNPTG)	NM_032520.3	0.517	1.67e-08
211898	POU domain, class 3, transcription factor 2 (POU3F2)	NM_005604.2	0.643	3.56e-08
209731	Ferritin heavy-chain (FTH1)	NM_213975.1	0.595	3.56e-08
214602	Acetyl-Coenzyme A carboxylase alpha (ACACA), transcript variant 2	NM_198839.1	0.473	6.06e-08
100718	Adenylate kinase 3 (AK3)	NM_016282.2	0.445	6.06e-08
213748	Hypothetical protein LOC284106 (LOC284106)	XM_375449.1	0.304	7.54e-08
216045	Phosphodiesterase 4D, cAMP-specific (PDE4D)	NM_006203.3	0.670	7.54e-08
218317	Ferritin heavy-chain (FTH1)	NM_213975.1	0.332	8.53e-08
211683	Ferritin heavy-chain (FTH1)	NM_213975.1	0.537	9.79e-08
202380	Protein phosphatase 1, regulatory subunit 2 pseudogene 3 (PPP1R2P3)	NR_002168.1	0.497	1.16e-07
103450	3-hydroxy-3-methylglutaryl-Coenzyme A synthase 1 (soluble) (HMGCS1)	NM_002130.4	0.387	1.16e-07
210738	Ferritin heavy-chain (FTH1)	NM_213975.1	0.538	1.65e-07
217101	Leucine zipper protein 1 (LUZP1)	NM_033631.2	0.741	1.84e-07
209872	Aldo-keto reductase family 1, member C4 (AKR1C4)	NM_001818.2	0.634	3.65e-07
101435	Muscle-specific intermediate filament desmin (LOC396725)	NM_001001535.1	0.559	4.57e-07
207680	Myelin expression factor 2 (MYEF2)	NM_016132.2	0.344	8.37e-07
103619	Glucan (1,4-alpha-), branching enzyme 1 (GBE1)	NM_000158.1	0.417	1.44e-06
205158	Glutathione S-transferase (MGST1)	NM_214300.1	0.447	1.59e-06
103416	Peroxiredoxin 3 (PRDX3), nuclear gene encoding mitochondrial protein	NM_014098.2	0.436	1.68e-06
210951	Ferritin heavy-chain (FTH1)	NM_213975.1	0.491	1.94e-06
214541	Homeo box (H6 family) 1 (HMX1)	NM_018942.1	0.334	2.17e-06
217401	Phosphate regulating gene (Phex)	NM_011077.1	0.592	2.82e-06
201622	Hypothetical protein LOC285016 (LOC285016)	NM_001002919.1	0.591	3.08e-06
217377			0.607	3.40e-06
217497	Similar to coronin, actin binding protein, 2B (LOC517030)	XM_595194.1	0.507	3.43e-06
101570	Sorting nexin 3 (SNX3), transcript variant 1	NM_003795.3	0.230	3.61e-06
216333	Brca1 associated protein 1 (Bap1)	NM_027088.1	0.297	3.74e-06
101369	Translocase of inner mitochondrial membrane 13 homolog (TIMM13)	NM_012458.2	0.333	3.98e-06
216897	Zinc finger protein 229 (ZNF229)	NM_014518.1	0.696	3.98e-06
201672			0.411	4.07e-06
100874	Ubiquitin-conjugating enzyme E2R 2 (UBE2R2)	NM_017811.3	-0.453	4.31e-06
217389	SMAD, mothers against DPP homolog 1 (Drosophila) (SMAD1)	NM_001003688.1	0.623	5.08e-06
103364	Sulfotransferase family, cytosolic, 2A, member 1 (SULT2A1)	NM_003167.2	0.325	5.08e-06
214446			0.484	5.08e-06
103263	Retinol dehydrogenase 12 (all-trans and 9-cis) (RDH12)	NM_152443.1	0.336	6.64e-06
220907	3-hydroxy-3-methylglutaryl-Coenzyme A synthase 1 (soluble) (HMGCS1)	NM_002130.4	0.363	7.29e-06
217413	Suppressor of cytokine signaling 5 (SOCS5), transcript variant 1	NM_014011.4	0.609	8.04e-06
209887			0.416	8.52e-06
211211	KIAA0999 protein (KIAA0999)	NM_025164.3	0.284	9.46e-06
104110	Cytochrome P450 51 (CYP51)	NM_214432.1	0.365	9.98e-06
204992			0.381	1.12e-05
100545	Isopentenyl-diphosphate delta isomerase (IDI1)	NM_004508.2	0.365	1.38e-05
100618	Myelin basic protein (MBP)	NM_001001546.1	0.272	1.38e-05
104176	Aminolevulinate, delta-, synthase 1 (ALAS1), transcript variant 1	NM_000688.4	0.407	1.42e-05
211043	Testis expressed sequence 261 (TEX261)	NM_144582.2	0.390	1.42e-05
209191	Chromosome 20 open reading frame 50 (C20orf50)	XM_046437.7	0.416	1.42e-05
221311			0.331	1.42e-05
201823	Cytochrome b-5 (CYB5)	NM_001001770.1	0.439	1.66e-05
209002	Malic enzyme 2, NAD(+)-dependent, mitochondrial (ME2)	NM_002396.3	-0.232	1.66e-05
212725	Immunoglobulin superfamily, member 8 (IGSF8)	NM_052868.1	0.181	1.89e-05
203290	Hypothetical protein MGC33214 (MGC33214)	NM_153354.2	-0.268	2.10e-05
103537	Regulatory factor X, 2 (influences HLA class II expression) (RFX2)	NM_000635.2	0.495	2.16e-05
211919			0.430	2.16e-05
220629	Chromosome 6 open reading frame 89 (C6orf89)	gi|47271470|ref|	-0.451	2.16e-05
200365	HDCMA18P protein (HDCMA18P)	NM_016648.1	-0.342	2.23e-05
207244	Superoxide dismutase 1, soluble (SOD1)	NM_000454.4	0.267	2.26e-05
102085	Steroid membrane binding protein (PGRMC1)	NM_213911.1	0.265	2.33e-05
103751	Glutathione peroxidase 4 (GPX4)	NM_214407.1	0.447	2.34e-05
204615	Solute carrier organic anion transporter family, member 1B3 (SLCO1B3)	NM_019844.1	0.297	2.65e-05
102131	Integral membrane protein 2B (ITM2B)	NM_021999.2	0.327	2.95e-05
100094	Poly(A) binding protein, cytoplasmic 1 (PABPC1)	NM_002568.3	0.375	3.07e-05
201085			0.344	3.16e-05
219913	Similar to Probable RNA-dependent helicase p68 (LOC505151)	XM_581395.1	0.418	3.21e-05
215566	Protein phosphatase 4 (formerly X), catalytic subunit (PPP4C)	NM_002720.1	0.330	3.37e-05
216418	Similar to omega protein (LOC91353)	NM_001013618.1	0.240	3.54e-05
215949	Hypothetical LOC400120 (LOC400120)	NM_203451.1	0.604	3.56e-05
103431	C-myc binding protein (MYCBP)	NM_012333.3	0.236	3.98e-05
101450			0.306	4.34e-05
209773	Similar to protein RAKc (LOC439947)	XM_495795.1	0.462	4.46e-05
105008	Cytochrome P450 19A2 (CYP19A2)	NM_214430.1	0.418	5.57e-05
103550	Phosphoenolpyruvate carboxykinase 1 (soluble) (PCK1)	NM_002591.2	-0.299	5.57e-05
206124	Solute carrier family 24, member 5 (SLC24A5)	NM_205850.1	-0.211	5.96e-05
212000	Upstream of NRAS (UNR), transcript variant 2	NM_007158.4	0.239	5.96e-05
214660	HSPC038 protein (LOC51123)	NM_016096.2	0.232	6.01e-05

Gene profiling was done using 30 arrays and analysed using linear models. Fold change (M-values) and statistical significance (FDR-adjusted p-values) is shown. The clone names refer to hits to human, mouse or pig genes. Some genes are represented by several different clones on the array and may therefore show up more than once in the table, while some have no hits to the abovementioned species.

**Table 2:** Top 100 genes identified in Norwegian Landrace

ID	Name	gene_id	M	adj.P.Val
214052	Carbonyl reductase/NADP-retinol dehydrogenase (DHRS4)	NM_214019.1	1.106	1.94e-08
213358	Microtubule-associated protein 1 light chain 3 alpha (MAP1LC3A)	NM_032514.2	0.805	2.41e-08
210231	Ferritin, light polypeptide (FTL)	NM_000146.3	0.884	2.49e-08
103463	Cytochrome P450 17A1 (CYP17A1)	NM_214428.1	1.278	3.06e-08
209574	KIAA1423 (KIAA1423)	XM_376550.2	0.466	3.06e-08
215500	Ferritin, light polypeptide (FTL)	NM_000146.3	0.820	3.27e-08
210851	Ferritin, light polypeptide (FTL)	NM_000146.3	0.910	4.39e-08
211570	Similar to hypothetical protein (LOC475155)	XM_532385.1	0.488	9.99e-08
217877	Ferritin, light polypeptide (FTL)	NM_000146.3	0.838	1.76e-07
218005			0.902	1.82e-07
220047	Cell division cycle associated 7 (CDCA7), transcript variant 1	NM_031942.3	-0.524	2.21e-07
203529	Cystatin F (leukocystatin) (CST7)	NM_003650.2	0.727	3.30e-07
211448	Golgi phosphoprotein 3-like (GOLPH3L)	NM_018178.3	0.778	4.07e-07
213434	Eukaryotic translation elongation factor 1 alpha 1 (EEF1A1)	NM_001402.4	0.884	4.76e-07
213869	PRA1 domain family, member 2 (PRAF2)	NM_007213.1	0.258	1.79e-06
105119	Steroidogenic acute regulatory protein (STAR)	NM_213755.2	0.378	1.79e-06
100252	Ras homolog gene family, member Q (Rhoq)	NM_053522.1	0.330	4.40e-06
209883			0.442	7.35e-06
211898	POU domain, class 3, transcription factor 2 (POU3F2)	NM_005604.2	0.541	7.81e-06
102025	Cytochrome b-5 (CYB5)	NM_001001770.1	0.397	8.88e-06
209731	Ferritin heavy-chain (FTH1)	NM_213975.1	0.421	2.15e-05
201622	Hypothetical protein LOC285016 (LOC285016)	NM_001002919.1	0.602	2.60e-05
101322	Ferredoxin (FDX1)	NM_214065.1	0.420	2.69e-05
206784	Ferritin heavy-chain (FTH1)	NM_213975.1	0.345	4.90e-05
209887			0.378	5.19e-05
215969	N-acetylglucosamine-1-phosphotransferase, gamma subunit (GNPTG)	NM_032520.3	0.362	5.50e-05
211014	Elastin microfibril interfacer 2 (EMILIN2)	NM_032048.2	0.235	5.50e-05
211919			0.393	5.50e-05
210951	Ferritin heavy-chain (FTH1)	NM_213975.1	0.401	6.77e-05
105008	Cytochrome P450 19A2 (CYP19A2)	NM_214430.1	0.338	8.16e-05
210738	Ferritin heavy-chain (FTH1)	NM_213975.1	0.424	8.16e-05
201823	Cytochrome b-5 (CYB5)	NM_001001770.1	0.454	8.16e-05
100826	Chromosome 15 open reading frame 24 (C15orf24)	NM_020154.2	-0.230	8.39e-05
101872	Anthrax toxin receptor 1 (ANTXR1), transcript variant 1	NM_032208.1	0.339	8.39e-05
211040	Cytochrome P450 11A1 (CYP11A1)	NM_214427.1	0.479	9.63e-05
103622	Chaperonin containing TCP1, subunit 4 (delta) (CCT4)	NM_006430.2	0.222	0.00010
211683	Ferritin heavy-chain (FTH1)	NM_213975.1	0.390	0.00010
209678	Parvin, gamma (PARVG)	NM_022141.4	0.354	0.00012
211043	Testis expressed sequence 261 (TEX261)	NM_144582.2	0.321	0.00014
100650	Brain expressed, X-linked 1 (BEX1)	NM_018476.2	0.410	0.00017
102207	Similar to RUN and FYVE domain-containing 2 (LOC441022)	XM_496700.1	0.322	0.00019
202502	A disintegrin and metalloproteinase domain 5 (ADAM5)	NR_001448.1	0.265	0.00019
221253	Adaptor-related protein complex 1, mu 2 subunit (AP1M2)	NM_005498.3	0.277	0.00019
210077	Cytochrome P450 2C49 (CYP2C49)	NM_214420.1	0.211	0.00020
201085			0.288	0.00021
211785	Zinc finger, DHHC domain containing 14 (ZDHHC14)	NM_153746.1	0.346	0.00023
104944	GTP cyclohydrolase 1 (dopa-responsive dystonia) (GCH1)	NM_000161.1	-0.593	0.00024
201672			0.293	0.00026
102125	Hypothetical protein HSPC138 (HSPC138)	NM_016401.2	0.209	0.00026
211821	Phosphatidylserine receptor (PTDSR)	NM_015167.1	0.726	0.00033
204992			0.415	0.00036
202380	Protein phosphatase 1, regulatory subunit 2 pseudogene 3 (PPP1R2P3)	NR_002168.1	0.422	0.00037
213748	Hypothetical protein LOC284106 (LOC284106)	XM_375449.1	0.221	0.00037
103177	Cytochrome b-5 (CYB5)	NM_001001770.1	0.566	0.00037
103075	Emopamil binding protein (sterol isomerase) (EBP)	NM_006579.1	0.227	0.00038
210827	Membrane-associated protein 17 (MAP17)	NM_001001769.1	0.269	0.00039
213497	NADH dehydrogenase (ubiquinone) 1, 1, 6kDa (NDUFC1)	NM_002494.2	0.271	0.00039
104615	Superoxide dismutase 1, soluble (SOD1)	NM_000454.4	0.268	0.00040
103619	Glucan (1,4-alpha-), branching enzyme 1 (GBE1)	NM_000158.1	0.291	0.00046
214976	Cyclin M2 (CNNM2), transcript variant 1	NM_017649.3	0.181	0.00049
216693	Calcium/calmodulin-dependent protein kinase II beta (CAMK2B)	NM_172081.1	-0.404	0.00052
101799	Sarcolemma associated protein (SLMAP)	NM_007159.2	0.298	0.00052
100935	ADP-ribosylation-like factor 6 interacting protein 6 (ARL6IP6)	NM_152522.2	0.246	0.00053
203697	Hypothetical protein MGC40579 (MGC40579)	NM_152776.1	0.394	0.00055
101031	RIKEN cDNA B230380D07 gene (B230380D07Rik)	NM_172772.1	0.354	0.00055
209872	Aldo-keto reductase family 1, member C4 (AKR1C4)	NM_001818.2	0.634	0.00057
102689	Sulfotransferase family, cytosolic, 2A (SULT2A1)	NM_003167.2	0.326	0.00060
214972	Similar to Bax inhibitor-1 (BI-1) (LOC451883)	XM_509049.1	0.269	0.00060
209191	Chromosome 20 open reading frame 50 (C20orf50)	XM_046437.7	0.342	0.00062
101010	NADH dehydrogenase (ubiquinone) flavoprotein 1, 51kDa (NDUFV1)	NM_007103.2	0.195	0.00069
217625	Leucine rich repeat neuronal 1 (LRRN1)	NM_020873.3	0.235	0.00070
100267	Transducer of ERBB2, 1 (TOB1)	NM_005749.2	0.252	0.00070
204605	Maltase-glucoamylase (alpha-glucosidase) (MGAM)	NM_004668.1	-0.273	0.00070
100575	17beta-estradiol dehydrogenase (HSD17B4)	NM_214306.1	0.262	0.00070
104294	Sarcoglycan, epsilon (SGCE)	NM_003919.1	0.223	0.00075
214602	Acetyl-Coenzyme A carboxylase alpha (ACACA), transcript variant 2	NM_198839.1	0.294	0.00085
100781	Protein kinase, AMP-activated, beta 2 non-catalytic subunit (PRKAB2)	NM_005399.3	0.267	0.00090
204606			-0.229	0.00091
100545	Isopentenyl-diphosphate delta isomerase (IDI1)	NM_004508.2	0.266	0.00111
210439	FK506 binding protein 1A, 12kDa (FKBP1A), transcript variant 12B	NM_000801.2	0.233	0.00112
101524	Alpha-1,3-galactosyltransferase (GGTA1)	NM_213810.1	0.274	0.00120
103263	Retinol dehydrogenase 12 (all-trans and 9-cis) (RDH12)	NM_152443.1	0.297	0.00124
201437	Hypothetical protein MGC40489 (MGC40489)	XM_373742.3	-0.239	0.00124
219641	Ras association (RalGDS/AF-6) domain family 4 (RASSF4)	NM_032023.3	0.294	0.00124
218092	Procollagen C-endopeptidase enhancer (PCOLCE)	NM_002593.2	0.330	0.00124
214348	Glycerol-3-phosphate dehydrogenase 1-like (GPD1L)	NM_015141.2	0.205	0.00133
210245	Complement component 1, r subcomponent (C1R)	NM_001733.2	0.221	0.00136
209875	Scavenger receptor class B member 1 (SCARB1)	NM_213967.1	0.253	0.00145
105364	Tumor necrosis factor, alpha-induced protein 6 (TNFAIP6)	NM_007115.2	0.241	0.00147
212682	Sulfotransferase family, cytosolic, 2B, member 1 (SULT2B1)	NM_177973.1	0.206	0.00149
102760			0.310	0.00152
101913	Heat shock 60kDa protein 1 (chaperonin) (HSPD1)	NM_002156.4	0.265	0.00154
214601	Serine/threonine kinase 19 (STK19), transcript variant 1	NM_004197.1	0.298	0.00160
101632	Nuclear receptor coactivator 2 (NCOA2)	NM_006540.1	0.287	0.00160
210848	Annexin A7 (ANXA7), transcript variant 1	NM_001156.2	0.182	0.00160
221285	Serine (or cysteine) proteinase inhibitor, clade B member 3 (SERPINB3)	NM_006919.1	0.225	0.00160
219203	Peroxiredoxin 2 (PRDX2), nuclear gene encoding mitochondrial protein	NM_005809.4	0.325	0.00170
216273			0.257	0.00185
217065	Endonuclease G-like 1 (ENDOGL1)	NM_005107.1	-0.364	0.00185
211482	Ubiquitin-conjugating enzyme E2R 2 (UBE2R2)	NM_017811.3	0.229	0.00196

Gene profiling was done using 30 arrays and analysed using linear models. Fold change (M-values) and statistical significance (FDR-adjusted p-values) is shown. The clone names refer to hits to human, mouse or pig genes. Some genes are represented by several different clones on the array and may therefore show up more than once in the table, while some have no hits to the abovementioned species.

**Figure 1:**
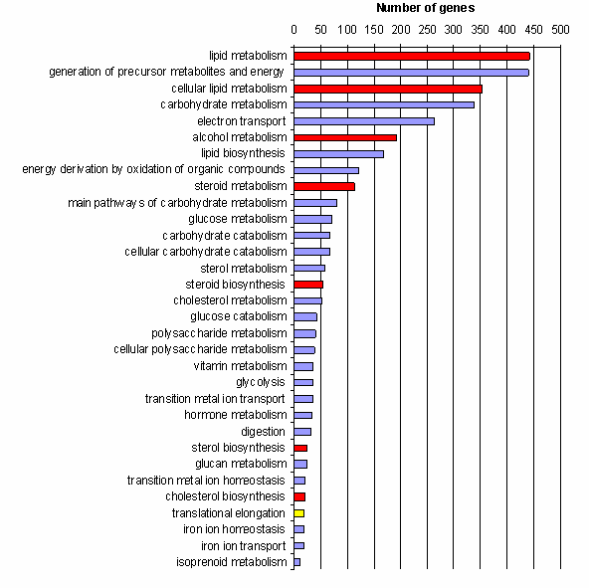
**Gene ontology (GO) results for the biological process ontology.** The genes differentially expressed at p < 0.01 were analysed for over-represented GO terms in the biological process ontology. Some terms were common for Duroc and Norwegian Landrace (red) while some were only significant in Duroc (yellow) or Norwegian Landrace (blue). Number of genes is the number of genes that was found significantly differentially expressed (p < 0.01) for a term.

**Figure 2:**
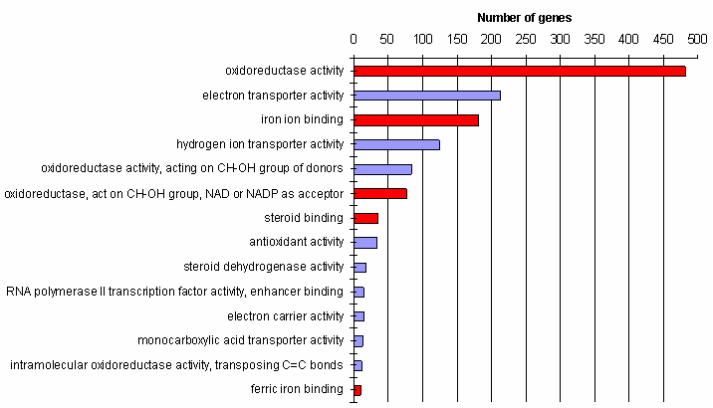
**Gene ontology (GO) results for the molecular function ontology.** The genes differentially expressed at p < 0.01 were analysed for over-represented GO terms in the molecular function ontology. Some terms were common for Duroc and Norwegian Landrace (red) while some were only significant in Norwegian Landrace (blue). Number of genes is the number of genes that was found significantly differentially expressed (p < 0.01) for a term.

The cDNA clones found to be differentially expressed were used to search for statistically overrepresented gene ontology (GO) terms compared with the GOs represented by all the genes on the array. The most significant terms analysed for molecular function in D were ferric iron binding, iron ion binding, oxidoreductase activity and steroid binding (Figure 1). For NL we also found terms related to functions like electron transport and steroid dehydrogenase activity (Figure 1). Furthermore, the genes were classified according to their biological processes (Figure 2) and cellular components (See Additional file 7: Gene ontology (GO) results for the cellular component ontology, Duroc and Additional file 8: Gene ontology (GO) results for the cellular component ontology, Norwegian Landrace). The biological processes most significant in both breeds were steroid biosynthesis and steroid metabolism.

Both real competitive PCR (rcPCR) and real-time PCR were used to validate expression profiles for ten of the interesting differentially expressed genes from the microarray experiment. Real-time PCR was used to confirm the expression of *CYP17*, *CYB5*, ferritin light polypeptide (*FTL*) and translation elongation factor 1 alpha 1 (*EEF1A1*) in the D samples. The NL pigs were not included in this study. The expression levels of all genes were normalized to β-actin (*ACTB*) and 18S rRNA. Three of the four genes, *FTL*, *CYB5 *and *CYP17*, were confirmed by this method, while for *EEF1A1 *no significant difference was found (Table 3). rcPCR was used to analyse gene expression profiles of sphingomyelin phosphodiesterase 1 (*SMPD1*), steroidogenic acute regulatory protein (*StAR*), sulfotransferase family 2A, dehydroepiandrosterone-preferring, member 1 (*SULT2A1*), 17-beta-hydroxysteroid dehydrogenase IV (*HSD17B4*), aldo-keto reductase family 1, member C4 (*AKR1C4*), and cytochrome P450, subfamily XIA, polypeptide 1 (*CYP11A1*). The expression levels of all genes were normalised using the housekeeping gene hypoxanthine guanine phosphoribosyltransferase 1 (*HPRT*). *StAR*, *SULT2A1*, *HSD17B4*, *AKR1C4 *and *CYP11A1 *were all differentially expressed in both D and NL (p < 0.01), while *SMPD1 *was not significantly differentially expressed in either of the two breeds (Table 4 and 5, respectively).

**Table 3:** Results from Real-time PCR

Gene	Real-time PCR fold change	
	
	ACTB	18S
CYP17A1	2.28 (p = 0.0015)	2.87 (p = 0.001)
CYB5	2.24 (p = 0.08)	2.84 (p = 0.025)
FTL	1.88 (p = 0.078)	2.58 (p = 0.0026)
EEF1A1	0.95 (p = 0.77)	1.30 (p = 0.23)

Real-time PCR was used to confirm expression variations of selected genes from the microarray study. Normalisation of the values was performed by using ACTB and 18S as control genes. Three of four examined genes were verified and one was contradicted.

**Table 4:** Results from the rcPCR bootstrap statistics (x4000), Duroc

Gene	Fold	Log10 Fold change	Bias	Std error	P value
SMPD1	1.31	0.12	0.0008	0.09	0.097
StAR	5.4	0.73	0.0004	0.10	0.000
SULT2A1	2.3	0.37	0.0007	0.08	0.000
HSD17B4	1.7	0.24	-0.0009	0.09	0.006
AKR1C4	1.7	0.23	0.0013	0.09	0.003
CYP11A1	2.4	0.38	0.0000	0.09	0.000

rcPCR was used to confirm expression variations of selected genes from the microarray study. Fold changes are calculated relative to baseline, which is the group of low androstenone (DL) in this case, and adjusted for the housekeeping gene HPRT. Five of six examined genes were verified and one was contradicted.

**Table 5:** Results from the rcPCR bootstrap statistics (x4000), Landrace

Gene	Fold	Log10 Fold change	Bias	Std error	P value
SMPD1	1.4	0.16	-1.60	0.18	0.185
StAR	12.8	1.11	-2.65	0.13	0.000
SULT2A1	2.4	0.38	-4.37	0.11	0.002
HSD17B4	1.7	0.23	-2.13	0.09	0.006
AKR1C4	2.5	0.39	-2.07	0.09	0.000
CYP11A1	3.2	0.50	5.84	0.12	0.000

rcPCR was used to confirm expression variations of selected genes from the microarray study. Fold changes are calculated relative to baseline, which is the group of low androstenone (NLL) in this case, and adjusted for the housekeeping gene HPRT. Five of six examined genes were verified and one was contradicted.

## Discussion

The present study describes the transcriptional profiles of testicle from animals with extreme high and low levels of androstenone in D and NL boars. Some of these genes have already been shown to affect levels of androstenone, although most of them have not previously been described in this context. Both *CYB5 *and *CYP17 *were up-regulated in both D high (DH) and NL high (NLH) animals and this was confirmed by real-time PCR. Over-expression of *CYB5 *cDNA clones in high androstenone animals agrees with results previously seen in protein expression studies [13,14]. Earlier studies on *CYP17 *have shown that polymorphisms in the coding region of the gene are not associated with androstenone levels [15], and that levels of the CYP17 protein are not correlated to levels of 16-androstene steroids in fat [14]. Notably, up-regulation of the gene expression of *CYP17 *in this study may be explained by its direct interaction with *CYB5 *in the andien-β synthase enzyme system. Differential expression of both genes was confirmed by real-time PCR in the D boars of our study.

StAR is involved in the transport of cholesterol from the outer to the inner mitochondrial membrane, where CYP11A1 converts cholesterol to pregnenolone. This has been described as the rate-limiting step in steroid hormone synthesis, after which pregnenolone may exit the mitochondria and can be metabolised to steroid hormones [21]. The cholesterol side chain cleavage enzyme system contains ferredoxin (FDX1) which functions as an electron transport system, transferring electrons from NADPH-dependent ferredoxin reductase to CYP11A1 [22,23]. *StAR *was over-expressed in NLH, while *FDX1 *and *CYP11A1 *were over-expressed in both the DH and NLH groups. Our rcPCR results show that both *StAR *and *CYP11A1 *are highly up-regulated in the DH and NLH groups, even though the microarray study was not able to detect differences in the gene expression of *StAR *in DH. Based on results from the rcPCR study, *StAR *seems to particularly affect the androstenone level since this gene is up-regulated as much as five times in DH pigs and twelve times in NLH pigs. Emopamil-binding protein (EBP), sterol-C4-methyl oxidase-like isoform 1 (SC4MOL) and the cytochrome P450 enzyme lanosterol 14-alpha-demetylase (CYP51) are enzymes involved in the cholesterol biosynthesis pathway [24-26]. *EBP *was up-regulated in NLH, while *SC4MOL *and *CYP51 *were up-regulated in the DH. Isopentenyl-diphosphate delta isomerase (IDI1) catalyses the inter-conversion of isopentenyl diphosphate (IPP) to dimethylallyl diphosphate (DMAPP), which is the substrate for the reaction that ultimately results in cholesterol, and this gene was up-regulated in both DH and NLH. The over-expression of all these genes may indicate that this early stage of steroid hormone biosynthesis is important for the production levels of androstenone. However, since cholesterol ultimately metabolises into both androstenone and testosterone, it might be impossible to use these genes as markers for low androstenone without simultaneously affecting levels of testosterone. However, further studies are needed to address this hypothesis.

In addition to FDX1, two additional ferritin cDNA clones were significantly up-regulated in the NLH and DH groups: *FTL *and the ferritin heavy-chain (*FTH1*). The differential expression of *FTL *was also confirmed by real-time PCR. Ferritin is an iron storage protein and plays a central role in numerous essential cellular functions [27]. Ferritin may also affect levels of androstenone through the CYB5/CYP450 electron transfer as the haem-containing cytochrome P450s need to receive electrons, e.g. from interaction with CYB5 [28], to be activated. 5-aminolevulinate synthase (ALAS1) is the first and rate-limiting enzyme in the haem biosynthesis pathway, providing haem for e.g. CYP450s [29]. This gene is up-regulated in DH. The gene ontology results also suggest an important role for ferric iron/iron ion binding for the levels of androstenone in adipose tissue.

Cytochrome P450 c19 (*CYP19*) encodes the enzyme aromatase, which catalyses the synthesis of estrogens from androgens. The pig secretes unusually high levels of estrogens from the testes [30] and is the only mammal known to express functionally distinct isoforms of this gene [31]. Our results show an up-regulation of *CYP19A2 *isoform in both NLH and DH boars, whereas the *CYP19A1 *isoform was up-regulated in DH. Estrogens are shown to be positively correlated with androstenone levels in fat [32] and estrone sulfate levels in plasma have been found positively correlated with both plasma and fat levels of androstenone [33]. SMPD1 is involved in the conversion of sphingomyelin to ceramide, which has been shown to inhibit CYP19 activity through induction of transcription factors [34]. *SMPD1 *was down-regulated in DH animals in the microarray study, but we were not able to confirm this in the rcPCR analyses.

*HSD17B4 *was found up-regulated for both DH and NLH, in both microarray and rcPCR analyses. The 17β-HSDs catalyse the last step in the formation of androgens and estrogens, and the HSD17B4 inactivates hormones very efficiently [35]. This has been reported for the substrates 17β-estradiol and 5-androstene-3β,17β-diol [36] but the enzyme also accepts other substrates [37]. The enzymes have previously been assigned to porcine Leydig and Sertoli cells [38] and porcine tissues express HSD17B4 as a predominant dehydrogenase [39]. Progesterone, a metabolite of pregnenolone, has been shown to increase porcine HSD17B4 activity [39] and the progesterone receptor membrane component 1 (*PGRMC1*) was over-expressed in DH. Another 17β-HSD, isoform 11, also called dehydrogenase/reductase family member 8 (*DHRS8*) was down-regulated in DH boars.

*AKR1C4 *belongs to the cytosolic aldo-keto reductases that act as 3α-/3β-/17β-/20α-hydroxysteroid dehydrogenases (HSDs) in human [40,41] and was highly up-regulated in DH and NLH pigs. This was also confirmed by rcPCR. Penning et al. [36] showed that all the isoforms AKR1C1-AKR1C4 could interconvert active androgens and oestrogens with their associated inactive metabolites, which prevents an excess of circulating steroid hormones and makes the steroids into substrates for conjugation reactions [40].

Nuclear receptor subfamily 5, group A, member 1 (*NR5A1*), also called steroidogenic factor 1 (SF-1), was over-expressed in DH boars. This transcription factor is involved in the regulation of numerous genes, including *StAR *[42], *CYP11A *[43], *CYP17 *[44] and *CYP19 *[45]. Also, nuclear receptor co-activator 4 (*NCOA4*), an androgen receptor (AR) activator often referred to as ARA70, was up-regulated in the DH group. The nuclear receptor co-activator 2 (*NCOA2*), another AR activator, was significantly over-expressed in NLH. Other genes regulating transcription and translation that were differentially expressed in this study include class III POU transcription factor (*POU3F2*), microtubule-associated protein light chain 3 isoform A (*MAP1LC3A*) and *EEF1A1 *which were up-regulated in the DH and NLH groups, eukaryotic translation elongation factor 1 beta (*EEF1B2*) which was up-regulated in DH boars, and nuclear receptor subfamily 3, group C, member 2 (*NR3C2*) which was down-regulated in DH pigs.

Conjugation reactions contribute to the levels and pattern of steroids present in the plasma circulation of the boar. The enzymes involved in conjugation reactions were first thought to strictly inactivate and eliminate the compounds by rendering them more water-soluble. However, the high levels of conjugated steroids that are present in the plasma of the boar may suggest that the biological role of these reactions is more complex [46,47]. A common family of conjugation enzymes is the sulfotransferases. *SULT2A1*, also called hydroxysteroid sulfotransferase (HST), was up-regulated in both DH and NLH boars in the microarray experiment and subsequently confirmed by rcPCR. SULT2A1 is a key sulfotransferase enzyme in terms of the 16-androstene steroids, and its activity in the testis is negatively correlated to androstenone concentrations in fat [19,48]. Sulfotransferase family 2 isoform B1 (SULT2B1) is also a HST enzyme, showing selectivity for the conjugation of 3β-hydroxysteroids [49]. The *SULT2B1 *was significantly up-regulated in NLH pigs. The over-expression of the sulfotransferase genes in this study does not correspond to the negative correlation to levels of androstenone previously reported for this enzyme family [48].

Another conjugation reaction is catalysed by the glutathione S-transferases (GSTs). They have been shown to bind hormones [50] and influence their transport, metabolism and action [51]. One isoform of these enzymes (GSTalpha) is shown to be active in the pig testis Leydig and Sertoli cells and positively regulated by both estradiol and testosterone [52]. Glutathione S-transferase omega (*GSTO1*) and glutathione S-transferase (*MGST1*) were over-expressed in DH. These enzymes have not previously been studied in connection with androstenone; however, no correlation was found between fat skatole levels and glutathione S-transferase activity [53].

D and NL show many similar molecular functions and biological processes in this study, although they also differ with respect to which genes are differentially expressed. [54]. This miscellany might be due to the generally higher androstenone levels observed in D compared to NL [8]. The variability in the potential for androstenone production or elimination may to some extent be explained by breed differences in age at sexual maturity [55]. Differences between D and NL have also been shown by differential expression of 3β-hydroxysteroid dehydrogenase and SULT2B1 proteins, both enzymes critical for steroid production [54]. The higher number of significant genes in D compared to NL may be explained by the more extreme androstenone values in this breed (See Additional file 9: Androstenone values), giving higher contrasts.

In addition to the genes listed, we also identified several highly differentially expressed cDNA clones with no homology to known human or mouse sequences. Further characterisation of these genes may uncover new and unexpected roles in association with androstenone. The over-expression of for example *AKR1C4*, *SULT2A1 *and *SULT2B1 *in high androstenone animals does not explain a role for these genes as inactivation enzymes and the role of these genes needs to be further studied in relation to androstenone levels. Additional studies of all the genes are necessary to see if their proteins show the same differential expression. Furthermore, identification of single nucleotide polymorphisms (SNPs) in the genes or in association with the genes can be used for breeding purposes. The exact function of genes from interesting gene ontology pathways, like ferric iron binding, iron ion binding, electron transport activities and oxidoreductase activities, also needs to be clarified.

## Conclusion

Our study detected differentially expressed genes that are previously found to affect androstenone levels in boars, as well as genes from pathways not formerly described in this aspect. We confirm the involvement of *CYP17 *and *CYB5 *and detect a number of other genes involved in the steroid hormone pathway that seem to be essential for androstenone levels. Besides *SULT2A1 *we identified other conjugation enzyme genes that might be important, including *SULT2B1*, *AKR1C4*, *GSTO1*, *MGST1 *and *HSD17B4*. The genetic profiles identified should be further examined to clarify their potential as molecular markers for reduced boar taint.

## Methods

### Animals

D and NL boars from NORSVIN's three boar testing stations were included in this study. The D and NL boars were on average 143 and 156 days at 100 kg live weight, respectively, and were slaughtered on average 14 days later. Tissue samples from testicle were frozen in liquid N_2 _and stored at -80°C until used for RNA isolation as described below. Fat samples were collected from the neck and stored at -20°C until used for androstenone measurements. Androstenone levels in fat were analysed by a modified time-resolved fluoroimmunoassay [56] using antiserum produced at the Norwegian School of Veterinary Science (NVH) [57]. A total of 1533 NL boars and 1027 D boars were analysed and statistical power calculations showed that selecting animals from each tail of the androstenone distribution was an effective way of obtaining high probability of finding differentially expressed genes with limited number of arrays. The power calculations suggested 30 arrays to be sufficient. Hence, for each breed, the 30 most extreme boars from each tail of the distribution were selected from all of the phenotyped animals (See Additional file 9: Androstenone values). In order to reduce family effects a maximum of two and three half sibs were chosen from NL and D, respectively. The same animals were used for verification of selected genes by rcPCR. Only D pigs were included in the real time PCR analysis.

### Expression profiling using microarrays

The microarrays (UltraGAPS coated slides, Corning Incorporated, MA, USA) were produced at the Faculty of Agricultural Sciences, Aarhus University and contained 27,774 cDNA clones printed in duplicates [58]. Out of these, 26,877 were PCR products amplified from cDNA clones and 867 were control DNA fragments. Of the 26.877 cDNAs, 21.417 map to 15.831 human gene transcript IDs corresponding to roughly 1.35 cDNAs per gene transcript. The remaining 5.460 cDNAs were thus estimated to cover around 4.036 gene transcripts yielding 19.867 gene transcripts in total. The annotation was done using NCBI Reference Sequence (RefSeq) database Release 11. The cDNA clones originated from the Sino-Danish sequencing project, covering 0.66× of the pig genome [59]. A more detailed description of the microarray can be found at the NCBI Gene Expression Omnibus (GEO [60]) database using the platform accession number GPL3608.

Total RNA for microarray studies and real-time PCR were extracted from testicle tissue using the RNeasy midi kit according to the manufacturer's instruction (Qiagen, CA, USA). Quantities were measured using a NanoDrop ND-1000 Spectrophotometer (NanoDrop Technologies, DE, USA) and qualities were examined by the 28S:18S rRNA ratio using the RNA 6000 Nano LabChip^® ^Kit on 2100 Bioanalyzer (Agilent Technologies, CA, USA). Aminoallyl-cDNA was synthesised from 20 μg of total RNA using the SuperScript indirect cDNA labelling system (Invitrogen Corporation, CA, USA) and labelled using ARES Alexa Fluor 488 or 594 labelling kit (Molecular Probes, OR, USA). Half of the samples were labelled with one dye and the other half with the other dye facilitating a direct balanced block hybridisation design, where the dye swap is balanced between the samples. As the animals are selected from a very large number of boars and the contrast between the two groups is extremely large, animals within each of the experimental group were treated as equal. We have prioritised a larger number of animals instead of hybridising all animals twice (with a dye swap), as we expect the biological variation to be greater than the technical variation. It may be noted that our experimental design eliminates the dye bias in the contrast 'high' versus 'low' androstenone, since SUM('high expressions')/30 minus SUM('low expressions')/30 is (30*H + 15*R + 15*G)/30 - (30*H + 15*R + 15*G)/30 = H - L, where H (L) denotes effect of high (low) androstenone, R (G) denotes the effect of red (green) dye and 30 is the number of arrays. Spike-in RNA from the Lucidea Universal ScoreCard (Amersham Biosciences) was added to the cDNA reactions. "Green" spike-in RNA was added to the samples labelled with Alexa-594 and "red" spike-in RNA was added to the samples labelled with Alexa-488. Purification of the amino-modified and fluorescently labelled cDNA was done using the NucleoSpin 96 Extract II PCR Clean-up kit (Macherey-Nagel, Düren, Germany), and a hybridization blocker (Invitrogen Corporation, CA, USA) containing polydA (Invitrogen Corporation, CA, USA) and Yeast tRNA (Invitrogen Corporation, CA, USA) was added. Each microarray was hybridised with one high and one low androstenone sample from the same breed, giving a total of 30 arrays for each breed. The high and low samples were paired randomly within each breed. Hybridisation was conducted in a Discovery XT hybridisation station (Ventana Discovery Systems, AZ, USA), followed by a manual wash and drying by centrifugation. More detailed descriptions of the microarray experiments are available at the GEO database through the series accession number GSE 7409.

The microarrays were scanned using a ScanArray Express scanner (Perkin Elmer Inc., MA, USA). Signal intensities were quantified using the ScanArray Express software and further analysis carried out in R version 2.2.1 [61] software package Linear Models for Microarray Analysis (Limma version 2.7.2) [62-64]. Mean foreground intensities were background corrected using the Edwards method [65] implemented in Limma using the median background intensities, and log-ratios were printtip loess normalised within arrays. The duplicate correlation function in Limma was used to consider the duplicate printing of each feature. As a quality check, MA-plots (M = log_2_594/log_2_488, A = (log_2_594 + log_2_488)/2), image plots and box plots were constructed using the Limma tools for visualisation of the data before and after normalisation. For assessing differential expression, Limma uses linear models in combination with an empirical Bayes method to moderate the standard errors of the estimated log-fold changes. The nominal p-values were corrected for multiple testing by false discovery rates (FDR) using Benjamini and Hochberg approach [66] and adjusted p-values < 0.01 were considered significant.

Since the Limma statistic provides a parametric test, it might be affected by outlier records. Therefore, we also conducted a more robust, but less powerful non-parametric test, namely Fisher's Sign Test (FST) [67].

The features of the arrays were mapped to a LocusLink identifier and an annotation package was built using the Bioconductor package AnnBuilder (version 1.9.14). Tests for significantly overrepresentation of GO terms (p < 0.01 and more than 10 significant genes) were conducted using the GOHyperG function of the Bioconductor package GOstats (version 1.6.0) [68,69].

### Quantitative rcPCR analysis and quantitative real-time RT-PCR analyses

A real competitive (rc) PCR gene expression analysis was used to verify some of the results from the microarray study. The method is build upon the MassARRAY methodology using the Quantitative Gene Expression (QGE) iPLEX system (Sequenom, CA, USA). Total RNA was isolated from testes by using the automatic DNA/RNA extractor (BioRobot M48 workstation; Qiagen; CA, USA) and first strand cDNA synthesis was conducted on 0.5 μg total RNA using SuperScript™-II Rnase H^- ^Reverse Transcriptase (Invitrogen, Carlsbad, CA, USA). Purified total RNA was treated with TURBO DNA-free™ (Ambion, Huntingdon, UK) for removal of contaminating DNA. Assay designs for the genes included in this study (See Additional file 10: Gene transcripts included in the rcPCR analyses) were multiplexed into a single reaction using MassARRAY QGE Assay Design software (Sequenom, CA, USA). The competitors, a synthetic DNA molecule that matched the sequence of the targeted cDNA region at all positions except for one single base served as an internal standard for each transcript. A 10-fold dilution of competitor was initially used over a wide range of concentrations to determine an approximate equivalence point, followed by a 7-fold dilution of competitor from 4.04 × 10^-11 ^to 1.43 × 10^-19 ^to achieve more accurate results. The cDNA and competitor were co-amplified in the same PCR reaction with the conditions 95°C for 15 minutes, 45 cycles each of 95°C for 20 second, 56°C for 30 seconds and 72°C for 1 minute, and finally 72°C for 3 minutes. After a clean-up step to remove unincorporated nucleotides, the PCR products served as templates for the primer extension reaction. The iPLEX reaction cocktail mix and PCR conditions were done as described in the Sequenom application guide [70]. Parallel PCR-reactions were performed for all samples and each of the products was printed with 2 replicates on a SpectroCHIP. The primer extension reaction generates distinct mass signals for competitor and cDNA-derived products, and mass spectrometric analysis generated signals from which the peak areas were calculated.

Because of collaborative reasons between two labs, another verification method used for the D boars was quantitative real-time PCR. Gene specific primers for four selected genes were generated from the Primer 3 software (See Additional file 11: Real-time PCR primers). Porcine *ACTB *and 18S rRNA amplifier set probes were used as endogenous control for normalisation. The gene *ACTB *was detected by Taqman probe whereas other genes were detected by SYBR Green probes. The same Duroc RNA samples as the ones used for the microarray experiments were applied. The RNA was synthesised into first-strand cDNA using SuperScript II Reverse Transcriptase (Invitrogen Corporation, CA, USA). The real-time PCR reaction was composed of 5 μL of Taqman master mix, 2 μL cDNA, 0.3 μL of forward and reverse primers (10 μM) and 0.05 μL of probe (10 μM). The real-time cycler conditions were 50°C for 2 minutes, 95°C for 10 minutes and 40 cycles each of 95°C for 15 seconds and annealing/extension at 60°C for 1 minute. Each reaction was conducted in triplicate on each individual sample with *ACTB *and 18S rRNA amplified as internal control genes. The real-time PCR amplification was performed using ABI PRISM 7900 HT sequence detection system. A cycle threshold value (C_T_) was recorded for each sample and a standard curve made from 4×, 2×, 1×, 0.5×, 0.125× and 0.0625× was used to calculate the relative mRNA levels.

In the rcPCR study, the gene expression levels of H and L androstenone boars were analysed using the software TITAN version 1.0–13 [71] that runs in the R statistical environment. The raw data from the Genotype Analyzer Software (Sequenom) was imported into TITAN and analysed using the default values of linear least squares polynomial regression with 4000 bootstrap replicates. The cDNA concentrations were corrected with respect to the housekeeping gene (HPRT), and p-values and confidence intervals for the fold changes were calculated. Details about the TITAN software are available at web [72].

Quantification of the real-time PCR amplification was performed using ABI PRISM 7900 HT sequence detection system. The standard curve method was used to calculate the relative mRNA levels.

## List of abbreviations

DH, Duroc High androstenone, NLH, Norwegian Landrace High androstenone, DL, Duroc Low androstenone, NLL, Norwegian Landrace Low androstenone, ACTB, β-actin, AKR1C1, Aldo-keto reductase family 1, member C1, AKR1C4, Aldo-keto reductase family 1, member C4, ALAS1, 5-aminolevulinate synthase, AR, androgen receptor, ARA70, androgen receptor, CYB5, Cytochrome b5, CYP11A1, Cytochrome P450, subfamily XIA, polypeptide 1, CYP17, Cytochrome P450 c17, CYP19, Cytochrome P450 c19, CYP51, Cytochrome P450 51, DHRS8, Dehydrogenase/reductase family member 8, DMAPP, Dimethylallyl diphosphate, EBP, Emopamit-binding protein, EEF1A1, Translation elongation factor 1 alpha 1, EEF1B2, Translation elongation factor 1 beta 2, FDX1, Ferredoxin, FTH1, Ferritin heavy-chain, FTL, Ferritin light polypeptide, FST, Fisher's sign test, GO, Gene ontology, GSTO1, Glutathione S-transferase omega, HPRT, Hypoxanthine guanine phosphoribosyltransferase 1, HSDs, Hydroxysteroid dehydrogenases, HSD17B4, 17-beta-Hydroxysteroid dehydrogenase IV, HST, Hydroxysteroid sulfotransferase, IDI1, Isopentenyl-diphosphate delta isomerase, IPP, Isopentenyl diphosphate, limma, linear models for microarray analysis, MAP1LC3A, Microtubule-associated protein light chain 3 isoform A, MGST1, Glutathione S-transferase, NCOA2, Nuclear receptor co-activator 2, NCOA4, Nuclear receptor co-activator 4, NR3C2, nuclear receptor subfamily 3, group C, member 2, NR5A1, Nuclear receptor subfamily 5 group A member 1, PGRMC1, Progesterone receptor membrane component 1, POU3F2, Class III POU transcription factor, rcPCR, real competitive PCR, SC4MOL, Sterol-C4-methyl oxidase isoform 1, SF-1, Steroidogenic factor 1, SMPD1, Sphingomyrlin phosphodiesterase 1, SNPs, single nucleotide polymorphisms, StAR, Steroidogenic acute regulatory protein, SULT, Sulfotransferase, SULT2A1, Sulfotransferase family 2A, dehydroepiandrosterone-preferring, member 1, SULT2B1, Sulfotransferase family 2A, dehydroepiandrosterone-preffering member 1

## Authors' contributions

MM carried out microarray molecular work, performed statistical analysis and drafted the paper. TM was involved in power calculations, performing statistical analysis and contributed to the paper. SL was involved in planning the project and writing the paper. CB was involved in planning the project and in charge of the lab facilities performing the microarray studies. XW carried out the real-time PCR work. LNC was involved in the microarray molecular work. IB was involved in statistical analysis. EG coordinated the study, was involved in planning the project, carried out rcPCR molecular work, and contributed to writing the paper. All authors have read and approved the final manuscript.

## Supplementary Material

Additional file 1 **Boxplot of the arrays**. Boxplots displaying the average log_2_-ratio distribution of raw background corrected log ratios and printtiploess normalised log ratios for the Duroc (a and c, respectively) and Norwegian Landrace (b and d, respectively) arrays. After within array normalisation, the log ratios were evenly distributed around 0, indicating no need for between array normalisation. Click here for file

Additional file 2 **Microarray results analysed using Limma, Duroc**. Gene profiling was done using 30 arrays and the cut-off for differentially expressed genes was a p-value of 0.01 after FDR correction. A positive t-statistics indicates up-regulation in animals with high androstenone values and vice versa. M-values are fold changes. The clone names are sequences with a hit to human, mouse or pig genes. Some genes are represented by several different clones on the array and may therefore show up more than once in the table, while some have no hits to the abovementioned species. Click here for file

Additional file 3 **Microarray results analysed using Limma, Norwegian Landrace**. Gene profiling was done using 30 arrays and the cut-off for differentially expressed genes was a p-value of 0.01 after FDR correction. A positive t-statistics indicates up-regulation in animals with high androstenone values and vice versa. M-values are fold changes. The clone names are sequences with a hit to human, mouse or pig genes. Some genes are represented by several different clones on the array and may therefore show up more than once in the table, while some have no hits to the abovementioned species. Click here for file

Additional file 4 **Genes common for Duroc and Norwegian Landrace at p < 0.01**. There were 72 genes in common for Duroc and Landrace at significance level p < 0.01. Click here for file

Additional file 5 **Microarray results analysed using Fisher's sign test, Duroc**. Gene profiling was done using 30 arrays and the cut-off for differentially expressed genes was a FDR of 0.01. A -1 indicates up-regulation, while a 1 indicates down-regulation in boars with high androstenone levels. The clone names are sequences with a hit to human, mouse or pig genes. Some genes are represented by several different clones on the array and may therefore show up more than once in the table, while some have no hits to the abovementioned species. Click here for file

Additional file 6 **Microarray results analysed using Fisher's sign test, Norwegian Landrace**. Gene profiling was done using 30 arrays and the cut-off for differentially expressed genes was a FDR of 0.05. A -1 indicates up-regulation, while a 1 indicates down-regulation in boars with high androstenone levels. The clone names are sequences with a hit to human, mouse or pig genes. Some genes are represented by several different clones on the array and may therefore show up more than once in the table, while some have no hits to the abovementioned species. Click here for file

Additional file 7 **Gene ontology (GO) results for the cellular component ontology, Duroc**. The genes differentially expressed at p < 0.01 in Duroc were analysed for over-represented GO terms in the cellular component ontology (p < 0.01). Click here for file

Additional file 8 **Gene ontology (GO) results for the cellular component ontology, Norwegian Landrace**. The genes differentially expressed at p < 0.01 in Duroc were analysed for over-represented GO terms in the cellular component ontology (p < 0.01). Click here for file

Additional file 9 **Androstenone values**. Animals were paired randomly between the high and low androstenone groups within each breed. The androstenone values (μg/g) in Duroc high (DH), Duroc low (DL), Norwegian Landrace high (NLH) and Norwegian Landrace low (NLL) animals are shown. Click here for file

Additional file 10 Gene transcripts included in the rcPCR analyses. Click here for file

Additional file 11 **Real-time PCR primers**. Primers designed to test four selected genes using quantitative real-time RT-PCR. Click here for file
