# Supplementary figures and images for: Gene expression profiles in testis of pigs with extreme high and low levels of androstenone
Source: BMC Genomics. 2007 Nov 7;8:405. doi: 10.1186/1471-2164-8-405 (PMC2204014; doi:10.1186/1471-2164-8-405)

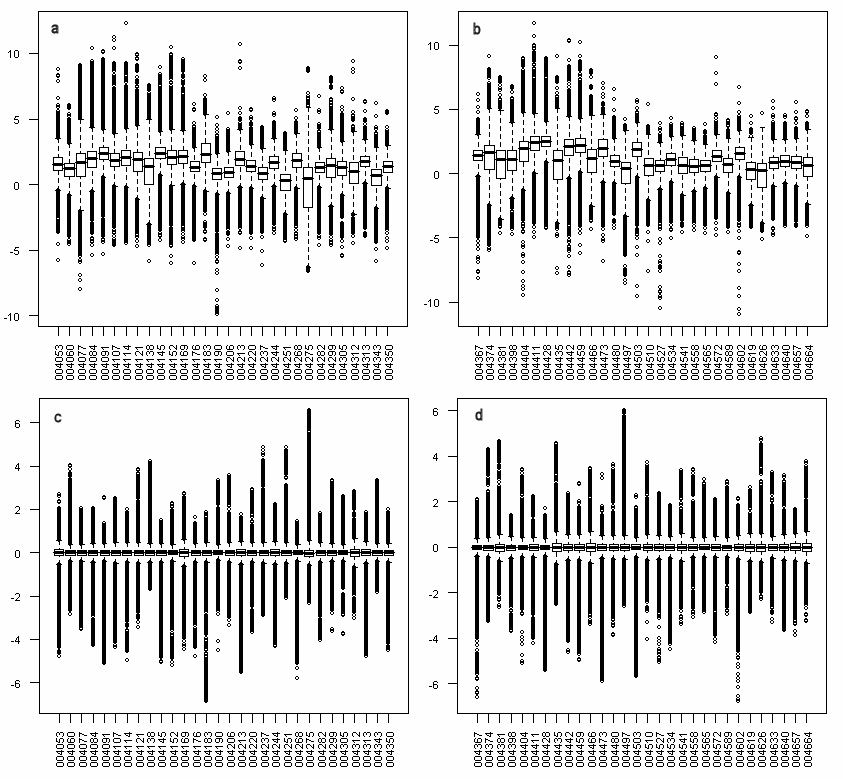

Supplement: Additional file 1 — Boxplot of the arrays. Boxplots displaying the average log2-ratio distribution of raw background corrected log ratios and printtiploess normalised log ratios for the Duroc (a and c, respectively) and Norwegian Landrace (b and d, respectively) arrays. After within array normalisation, the log ratios were evenly distributed around 0, indicating no need for between array normalisation. [file 1471-2164-8-405-S1.png]
