# Supplementary material for: Gene expression profiles in testis of pigs with extreme high and low levels of androstenone
Source: BMC Genomics. 2007 Nov 7;8:405. doi: 10.1186/1471-2164-8-405 (PMC2204014; doi:10.1186/1471-2164-8-405)
Supplement: Additional file 9 — Androstenone values. Animals were paired randomly between the high and low androstenone groups within each breed. The androstenone values (μg/g) in Duroc high (DH), Duroc low (DL), Norwegian Landrace high (NLH) and Norwegian Landrace low (NLL) animals are shown. [file 1471-2164-8-405-S9.doc]

| Slide ID | DH | DL | Slide ID | NLH | NLL |
| --- | --- | --- | --- | --- | --- |
| 13199854 | 9.1 | 0.5 | 13199520 | 4.77 | 0.16 |
| 13199853 | 15.33 | 0.47 | 13199068 | 5.71 | 0.19 |
| 13199029 | 12.51 | 0.17 | 13199069 | 4.15 | 0.18 |
| 13199028 | 9.43 | 0.47 | 13199070 | 4.55 | 0.07 |
| 13199026 | 8.76 | 0.48 | 13199071 | 3.88 | 0.15 |
| 13199025 | 8.61 | 0.51 | 13199072 | 7.77 | 0.16 |
| 13199024 | 9.76 | 0.54 | 13198960 | 6.86 | 0.17 |
| 13199183 | 20.01 | 0.44 | 13198961 | 7.02 | 0.17 |
| 13199184 | 9.77 | 0.45 | 13198962 | 13.89 | 0.14 |
| 13199185 | 9.2 | 0.48 | 13198963 | 5.5 | 0.2 |
| 13199186 | 16.92 | 0.39 | 13198964 | 5.39 | 0.09 |
| 13199187 | 8.77 | 0.33 | 13199588 | 4.26 | 0.15 |
| 13201862 | 9.01 | 0.01 | 13199589 | 3.95 | 0.18 |
| 13201861 | 8.97 | 0.53 | 13199592 | 4.83 | 0.2 |
| 13201860 | 9.65 | 0.46 | 13199632 | 5.83 | 0.17 |
| 13201859 | 8.76 | 0.56 | 13199627 | 4.81 | 0.08 |
| 13201858 | 9.7 | 0.31 | 13199628 | 4.4 | 0.13 |
| 13199532 | 8.38 | 0.31 | 13199629 | 4.76 | 0.08 |
| 13199527 | 9.88 | 0.48 | 13199630 | 6.4 | 0.05 |
| 13199528 | 8.7 | 0.51 | 13202106 | 7.14 | 0.19 |
| 13199529 | 9.92 | 0.5 | 13202107 | 3.77 | 0.17 |
| 13199530 | 8.69 | 0.54 | 13202108 | 6.45 | 0.14 |
| 13199446 | 9.99 | 0.56 | 13202109 | 3.8 | 0.19 |
| 13199447 | 9.95 | 0.39 | 13202110 | 5.09 | 0.12 |
| 13199448 | 9.3 | 0.35 | 13202101 | 5.71 | 0.13 |
| 13199449 | 8.84 | 0.51 | 13202102 | 8 | 0.17 |
| 13199450 | 8.68 | 0.5 | 13202103 | 13.4 | 0.15 |
| 13199514 | 12.6 | 0.3 | 13202104 | 5.39 | 0.17 |
| 13199515 | 9.32 | 0.14 | 13202105 | 3.96 | 0.1 |
| 13199518 | 9.52 | 0.52 | 13202015 | 5.94 | 0.13 |
