# Supplementary material for: Gene expression profiles in testis of pigs with extreme high and low levels of androstenone
Source: BMC Genomics. 2007 Nov 7;8:405. doi: 10.1186/1471-2164-8-405 (PMC2204014; doi:10.1186/1471-2164-8-405)
Supplement: Additional file 10 — Gene transcripts included in the rcPCR analyses. [file 1471-2164-8-405-S10.doc]

| Gene | Accession number | Primer sequence |
| --- | --- | --- |
| SMPD1 | BP435032 | Forward 5’- ATATCATCGGCCGTATTCCC – 3’  Reverse 5’ – CTCGTACCTGTTCACGATTC – 3’  Extension primer 5’ – gCAGGGCACTGCCTGAAG - 3’ |
| StAR | NM_213755 | Forward 5’ – TTCCGTGTGTGTGCTGGCTG – 3’  Reverse 5’ – CGTGCTCAGCTCTGATGACCCCC – 3’  Extension primer 5’ – tCTCAGGCATCTCTCCAAAGTC – 3’ |
| SULT2A1 | DQ172907 | Forward 5’ – CCAAGGAAATGTGCCCTATGGAT – 3’  Reverse 5’ – GTATCAGGACGTTCTCCTTG – 3’  Extension primer5’ – tACATTCGTGGCTGGT– 3’ |
| HSD17B4 | X78201 | Forward 5’ – CCAAGAAACTGGAGACACTG - 3’  Reverse 5’ – AGATCTCCACCCTCAGATGGTAT – 3’  Extension primer 5’ – gAGTATCAGATGTTGGCACA – 3’ |
| AKR1C4 | NM_001038626 | Forward 5’- AGCTGGAGATGATCCTGAAC – 3’  Reverse 5’ – CATTCCACCTGGTTGCAGAC – 3’  Extension primer 5’ – AGACGGGCTTGTACT - 3’ |
| CYP11A1 | NM_214427 | Forward 5’ – GGAAATGACAAGCTGCTCTC – 3’  Reverse 5’ – ATGGATGTCGTGTCTACACC – 3’  Extension primer 5’ – CGGTAACATTGGCCTTA - 3’ |
| HPRT | AY609416 | Forward 5’ – CTGGCAAAACAATGCAAACC – 3’  Reverse 5’ – TTTCACCAGCAAGCTTGCAACCT – 3’  Extension 5’ – GCAAACCTTGCTTTCC - 3’ |
