# Supplementary material for: Gene expression profiles in testis of pigs with extreme high and low levels of androstenone
Source: BMC Genomics. 2007 Nov 7;8:405. doi: 10.1186/1471-2164-8-405 (PMC2204014; doi:10.1186/1471-2164-8-405)
Supplement: Additional file 11 — Real-time PCR primers. Primers designed to test four selected genes using quantitative real-time RT-PCR. [file 1471-2164-8-405-S11.doc]

| Gene | Sequence | Size (bp) |
| --- | --- | --- |
| 18S | Forward 5’ – CTCAACACGGGAAACCTCAC – 3’  Reverse 5’ – CGCTCCACCAACTAAGAACG – 3’  Probe SYBR Green 5’ – CCACCACC | 110 |
| ACTB | Forward 5’ – TTGGGCATGGAGTCCTGC – 3’  Reverse 5’ – CGCGATGATCTTGATCTTCATG – 3’  Probe FAM 5’ – GTGCGACGTGGACAT | 194 |
| CYP17A1 | Forward 5’ – ATCCCCCACAGGGCTATC – 3’  Reverse 5’ – CATCTGTGTCCTTGTCAATGGT – 3’  Probe SYBR Green 5’ – CTCCAGCA | 54 |
| CYB5 | Forward 5’ – TGCTCGAGAGTTGTCCAAAA – 3’  Reverse 5’ – GGCTTGGCAATCTTTGATCT – 3’  Probe SYBR Green 5’ – GGGAGCTG | 72 |
| FTL | Forward 5’ – TCTATTTCAACCGCGACGA – 3’  Reverse 5’ – CAAGAGACGCTCCGAACC – 3’  Probe SYBR Green 5’ – TGGCTCTG | 95 |
| EEF1A1 | Forward 5’ – AGAACATGATTACAGGCACTTCC – 3’  Reverse 5’ – TTCGAATTCACCAACACCAG – 3’  Probe SYBR Green 5’ – TGCTGTCC | 74 |
